# Canadian Consensus for Biomarker Testing and Treatment of TRK Fusion Cancer in Pediatric Patients

**DOI:** 10.3390/curroncol28010038

**Published:** 2021-01-09

**Authors:** Sébastien Perreault, Rose Chami, Rebecca J. Deyell, Dina El Demellawy, Benjamin Ellezam, Nada Jabado, Daniel A. Morgenstern, Aru Narendran, Poul H. B. Sorensen, Jonathan D. Wasserman, Stephen Yip

**Affiliations:** 1 Department of Neurosciences, Division of Child Neurology CHU Sainte-Justine, Montreal, QC H3T 1C5, Canada; 2 Department of Paediatric Laboratory Medicine, The Hospital for Sick Children, Toronto, ON M5G 1X8, Canada; rose.chami@sickkids.ca; 3 Department of Laboratory Medicine and Pathobiology, University of Toronto, Toronto, ON M5S 1A8, Canada; 4 Division of Pediatric Hematology, Oncology and Bone Marrow Transplant, British Columbia Children’s Hospital and Research Institute, Vancouver, BC V6H 3N1, Canada; rdeyell@cw.bc.ca; 5 Pathology Department, Children’s Hospital of Eastern Ontario, Ottawa, ON K1H 8L1, Canada; deldemellawy@cheo.on.ca; 6 Department of Pathology, Centre Hospitalier Universitaire Sainte-Justine, Université de Montréal, Montreal, QC H3T 1C5, Canada; benjamin.ellezam@umontreal.ca; 7 Department of Pediatric Hematology-Oncology, MUHC, Montreal, QC H4A 3J1, Canada; nada.jabado@mcgill.ca; 8 Division of Pediatric Hematology/Oncology, The Hospital for Sick Children, University of Toronto, Toronto, ON M5G 1X8, Canada; daniel.morgenstern@sickkids.ca; 9 Departments of Pediatrics, Oncology and, Biochemistry and Molecular Biology, Cumming School of Medicine, University of Calgary, Calgary, AB T2N 4N1, Canada; anarendr@ucalgary.ca; 10 Department of Pathology and Laboratory Medicine, University of British Columbia, Vancouver, BC V6T 1Z4, Canada; 11 Department of Molecular Oncology, BC Cancer, Vancouver, BC V5Z 1L3, Canada; phbsorensen@gmail.com; 12 Division of Endocrinology, Department of Pediatrics, Hospital for Sick Children, University of Toronto, Toronto, ON M5G 1X8, Canada; Jonathan.Wasserman@sickkids.ca

**Keywords:** *NTRK*, larotrectinib, entrectinib, targeted therapy, molecular testing, oncogenic drivers, TRK fusion, tumour-agnostic

## Abstract

Neurotrophic tyrosine receptor kinase gene fusions (*NTRK*) are oncogenic drivers present at a low frequency in most tumour types (<5%), and at a higher frequency (>80%) in a small number of rare tumours (e.g., infantile fibrosarcoma [IFS]) and considered mutually exclusive with other common oncogenic drivers. Health Canada recently approved two tyrosine receptor kinase (TRK) inhibitors, larotrectinib (for adults and children) and entrectinib (for adults), for the treatment of solid tumours harbouring *NTRK* gene fusions. In Phase I/II trials, these TRK inhibitors have demonstrated promising overall response rates and tolerability in patients with TRK fusion cancer who have exhausted other treatment options. In these studies, children appear to have similar responses and tolerability to adults. In this report, we provide a Canadian consensus on when and how to test for *NTRK* gene fusions and when to consider treatment with a TRK inhibitor for pediatric patients with solid tumours. We focus on three pediatric tumour types: non-rhabdomyosarcoma soft tissue sarcoma/unspecified spindle cell tumours including IFS, differentiated thyroid carcinoma, and glioma. We also propose a tumour-agnostic consensus based on the probability of the tumour harbouring an *NTRK* gene fusion. For children with locally advanced or metastatic TRK fusion cancer who have either failed upfront therapy or lack satisfactory treatment options, TRK inhibitor therapy should be considered.

## 1. Introduction

An oncogenic neurotrophic tyrosine receptor kinase 1 (*NTRK1*) gene fusion cloned from a colon cancer sample was first reported in 1982 based on murine fibroblast transformation assays [1,2]. However, the discovery of the *ETV6-NTRK3* gene fusion in infantile fibrosarcoma (IFS) in 1998 marked the first description of *NTRK* fusions as recurrent oncogenic drivers in human cancers [3,4]. The *NTRK1*, *NTRK2*, and *NTRK3* genes encode the TRKA, TRKB, and TRKC receptors, respectively [1,3,4]. Under physiological conditions, ligand-binding by NGF/NT3, BDNF/NT4, and NT3 at TRKA, TRKB, and TRKC receptors, respectively, induces homodimerization and signalling through downstream pathways [1,3,5,6,7,8,9,10]. These pathways play a role in multiple processes, including cell differentiation, pain signalling, thermoregulation, regulation of movement, memory, mood, appetite, body weight, and proprioception [1,3,5,6,7,8,9,10]. The 3′ sequences of *NTRK1*, *NTRK2*, or *NTRK3* genes, encoding the intracellular tyrosine kinase domain, fuse with a number of different 5′ partners [1]. Breakpoints most often occur in introns and maintain open reading frames of resulting fusion transcripts [11]. Encoded chimeric oncoproteins contain constitutively activated kinase domains, as the 5′ partner of the fusion contributes a protein domain that promotes ligand independent oligomerization (i.e., without requiring ligand binding), thus driving kinase activation and uncontrolled signaling and oncogenesis [1]. These fusions are typically mutually exclusive with other primary oncogenic drivers [12,13].

### 1.1. Targeted Therapy for TRK Fusion Cancer

Two agents are currently approved by Health Canada for treatment of TRK fusion cancer: larotrectinib (VITRAKVI, Bayer Inc. Mississauga, ON, Canada) and entrectinib (ROZLYTREK, Hoffmann-La Roche Limited, Mississauga, ON, Canada). Both were studied in tumour-agnostic, molecularly defined “basket trials,” as *NTRK* gene fusions are rare and large randomized Phase III trials in specific tumour types are not feasible [14,15,16]. Basket trials have been suggested as a necessary tool in the move toward an era of precision oncology [14].

Larotrectinib is a highly selective TRK inhibitor [15]. The following trials defined initial experiences [15,17,18]:

- Adult Phase I trial (NCT02122913),
- SCOUT (pediatric [≤21 years of age] Phase I/Phase II basket trial) (NCT02637687),
- NAVIGATE (adolescent/adult [≥12 years of age] Phase II basket trial) (NCT02576431).

The Phase I dose escalation trial included patients with metastatic solid tumours, regardless of *NTRK* gene fusion status, including patients with *NTRK* variants/substitutions and amplifications [17]. Since drug activity was only seen in the patients harbouring *NTRK* gene fusions, future studies focused on this gene fusion-positive patient population [17]. In a pooled analysis, at a data cut-off of February 2019, a total of 159 patients with locally advanced or metastatic TRK fusion-positive non-primary-central nervous system (CNS) solid tumours and a median of one prior systemic therapy (including 52 pediatric patients [<18 years of age]) were enrolled in these three trials [15]. This constitutes the largest and longest-term dataset for any TRK inhibitor to date [15]. The overall response rate (ORR) (complete plus partial response) for the total population was 79% and in the pediatric patients was 92% [15,19]. In the pediatric population (*n* = 59), the majority of whom had IFS (58%) or other soft tissue sarcomas (STSs) (36%), the median duration of response (DOR) was not estimable (NE; 95% confidence interval [CI] 21.2-NE), median progression-free survival (PFS) was NE (95% CI 22.1-NE), and median overall survival (OS) was NE (95% CI NE-NE) [19]. Twenty four patients (20 of whom were pediatric) with TRK fusion-positive primary CNS tumour were treated with larotrectinib in the SCOUT and NAVIGATE trials (13 high-grade glioma [HGG], 5 low-grade glioma [LGG], and 3 others) [20]. The ORR was 29%, median DOR was 4.9 months, median PFS was 11 months, and median OS was NE [20]. Similar to the experience in solid cancers, *NTRK* gene fusions do exist in a small percentage of hematological malignancies [21] and RNA-based fusion assays should be attempted on those without known oncological drivers [22]. However, there is currently no national consensus on *NTRK* gene fusion testing in this cohort.

The majority of adverse events (AEs) were Grade 1–2 [19]. Fifteen percent of patients reduced their dose due to AEs and 3% discontinued treatment due to AEs [19]. The most common larotrectinib-related serious AEs included elevated alanine aminotransferase (two of 260 patients [<1%] who received at least one dose of larotrectinib [safety population]), elevated aspartate aminotransferase (two [<1%]), and nausea (two [<1%]) [15].

Entrectinib is a multikinase TRK, ROS1, and ALK inhibitor [16]. It was studied in the following trials in which patients with locally advanced or metastatic TRK fusion-positive solid tumours were included [16,23]:

- ALKA-372-001 (adult Phase I basket trial) (NCT02097810),
- STARTRK-1 (adult Phase I basket trial) (NCT02097810),
- STARTRK-2 (adult Phase II basket trial) (NCT02568267),
- STARTRK-NG (adolescent/pediatric [≤20 years of age] Phase I/II basket trial) (NCT02650401).

In a pooled analysis at a data cut-off of May 2018, there were 54 evaluable adult patients with TRK fusion cancer with a median of one prior systemic therapy [16]. The ORR was 57%, median DOR was 10.4 months, median PFS was 11.2 months, and median OS was 21 months [16]. In STARTRK-NG, the majority of patients had neuroblastoma (43%), 23% of patients had HGG, and the rest had a variety of primary CNS or extracranial solid tumours [23]. The ORR in the fusion-positive pediatric patients (including those with primary CNS tumors) was 86% (*n* = 10 with *NTRK* gene fusions, *n* = 3 with ALK fusion and *n* = 4 with ROS1 fusions) [23]. 

In the pediatric safety population (*n* = 34), 32.4% of patients reduced their dose due to AEs and 8.8% of patients discontinued drug due to AEs [23]. Adverse events of special interest included weight gain, neurological effects (somnolence: seven [21%], paresthesia: two [6%], and ataxia: four [12%] all Grade 1/2), and fracture (seven [20.6%] of 34 patients in the safety population, all grades) [23]. 

### 1.2. Development of Resistance to TRK Inhibitors

Acquired mutations in *NTRK* that lead to mutations in the kinase domain of TRK interfere with the binding of first-generation TRK inhibitors and cause resistance [24,25,26]. Acquired mutations have been demonstrated to lead to resistance to both entrectinib and larotrectinib [24,25]. Second-generation TRK inhibitors such as selitrectinib, repotrectinib, and taletrectinib are currently in development to overcome known resistance mechanisms [24,25,27].

### 1.3. Regulatory and Funding Status of TRK Inhibitors in Canada, as of 2020

Larotrectinib was approved by Health Canada in July 2019 for adult and pediatric patients with solid tumours with *NTRK* gene fusions without a known acquired resistance mutation, whose disease is metastatic, or where surgery would result in severe morbidity, and who have no satisfactory alternate treatment options [28]. Entrectinib was approved by Health Canada in February 2020 for the treatment of adult patients with unresectable locally advanced or metastatic extracranial solid tumours, including brain metastases, that have an *NTRK* gene fusion without a known acquired resistance mutation, and with no satisfactory treatment options [29]. Entrectinib has not been approved for patients <18 years old in Canada, although it is approved for patients 12 years and older with TRK fusion cancer by the Food and Drug Administration in the United States [29,30].

### 1.4. NTRK Gene Fusion Testing

Given *NTRK* gene fusions are rare, the key issues with testing are how to identify all patients with TRK fusion cancer in the most time-, cost-, and tissue-efficient manner. We briefly discuss the pros and cons of various methods, focusing primarily on immunohistochemistry (IHC) and next generation sequencing (NGS), as these are the focus of the in the CANTRK Ring Study [31]. The CANTRK Ring Study is a Canadian multi-centre *NTRK* gene fusion validation study, which aims to harmonize and standardize Canadian molecular pathology laboratory approaches to testing [31]. It was designed to assist laboratories across the country to validate laboratory-developed IHC assays for pan-TRK screening as well as comprehensive molecular testing by NGS to detect *NTRK* gene fusions, relying on existing diagnostic laboratory infrastructure [31]. Other testing methods are also reasonable if validated and are outlined briefly. The type of specimen (formalin-fixed, paraffin-embedded [FFPE], fresh, tissue, cell-free DNA, etc.) will vary by tumour type and tests should be validated in all potential specimens and criteria for acceptability should be established [32].

#### 1.4.1. Immunohistochemistry

Pan-TRK IHC uses a recombinant rabbit monoclonal antibody (i.e., EPR17341) targeted to a common domain on TRKA, TRKB, and TRKC proteins [33]. Staining above background in at least 1% of tumour cells is considered positive [34]. While most *NTRK* gene fusion-positive cases show cytoplasmic staining, localization varies based on the fusion partner [35], and can be nuclear, cytoplasmic and membranous, or may show combined patterns. The nuclear staining pattern is characteristic and virtually diagnostic of the *ETV6-NTRK3* fusion. Since IHC detects both TRK wildtype and TRK fusion proteins, there is a risk of false positive results, and hence a confirmatory molecular test is required [36]. In tissues with endogenous basal wildtype TRK expression such as smooth muscle, testes, ovaries, and central and peripheral nervous system, IHC cannot discriminate between *NTRK* gene fusion products and the wildtype TRK protein [33,37,38,39]. Immunohistochemistry also cannot be used in CNS tumours due to basal wildtype TRK expression [33]. False negative IHC results have been reported in *NTRK3* gene fusions [40]. It is also important to use appropriate positive controls to exclude the possibility of a false negative due to improper specimen preparation, although in general, IHC has a high negative predictive value [36,41]. Pan-TRK IHC has demonstrated a sensitivity of 95.2% and a specificity of 100% across a range of predominantly non-CNS tumour types [33]. In another study, pan-TRK IHC specificity was low in breast carcinoma (82%) and salivary gland carcinoma (52%), and both specificity and sensitivity were poor in sarcoma [42]. Undifferentiated sarcomas with *YWHAE* and *BCOR* alterations can over-express TRKC, resulting in positive pan-TRK IHC [43].

There is some debate about the utility of pan-TRK IHC. Immunohistochemistry is widely implemented, with a quicker turnaround time and lower cost than molecular methods, and IHC contributes to a multimodal diagnosis [33]. However, a positive IHC test alone is insufficient to confirm a diagnosis of TRK fusion cancer and is not an indication for TRK inhibitor therapy. These must be tested with a molecular method to confirm/exclude *NTRK* gene fusions due to the suboptimal sensitivity and specificity of currently available pan-TRK assays. Additionally, TRK expression patterns in fusion-positive cancers are dependent on the exact nature of the fusion event and the specificity for IHC is largely dependent on the type of cell line/tumour tested [44]. However, judicious use and interpretation of pan-TRK IHC is recommended as a cost- and resource-efficient first-line screening tool in some specific tumour types such as STS with IFS-like morphology and particularly those with co-expression of S100 and CD34 immunostaining [45]. Nonetheless, given the comparatively small number of pediatric patients with cancer vs. adult patients, IHC may have a limited role in the pediatric setting. In some select pediatric cancer types, it may be feasible for all newly diagnosed patients to undergo molecular testing. 

#### 1.4.2. Fluorescence In-Situ Hybridization (FISH)

The *ETV6-NTRK3* fusion is pathognomonic for IFS and is routinely detected by a break-apart FISH assay at time of diagnosis, and we believe additional molecular testing is not required [35]. Similarly, canonical fusions in *ETV6-NTRK3* are also seen in cellular and mixed congenital mesoblastic nephroma, and FISH testing would routinely be sought to confirm this diagnosis [36,46,47,48]. The FISH test has the advantage of generally only requiring one unstained slide per probe and retaining single cell morphology of the interrogated population [49]. One should interpret negative results by FISH and NanoString assay with caution, particularly in cases with morphology and immunoprofile suspicious of *NTRK*-related tumours. There is a high false-negative rate with *NTRK1* gene fusions due to a high incidence of intrachromosomal fusions [35,49]. If the 5′ partner is unknown, three separate break apart FISH tests are required to detect *NTRK1*, *NTRK2*, and *NTRK3* fusions and intrachromosomal fusions are difficult to identify [35].

#### 1.4.3. Next-Generation Sequencing

Next generation sequencing can analyze DNA or RNA, has high sensitivity and specificity compared with other methods, and using multigene panels, can simultaneously assess multiple mutations/fusions [36,50]. The DNA or RNA can be extracted from FFPE tissue with as few as three FFPE slides or 10 ng of DNA or RNA [49]. The turnaround time for NGS is longer than IHC or FISH and requires more structural and analytical investment/infrastructure [35].

The RNA method is preferred for *NTRK* gene fusions because it only identifies in-frame, transcribed fusions at basepair resolution and can determine whether the protein would be translated [35,36,51]. It avoids difficulties of sequencing large intronic regions, particularly for *NTRK2* and *NTRK3* [35,36,50,51]. Lastly, fusion-specific NGS panels are optimally designed to detect such fusion events generated from a large permutations of *NTRK* gene families fusing with a large cohort of binding partners.

### 1.5. Access to NTRK Gene Fusion Testing in Canada

Pediatric patients may have access to molecular testing through national or institutional research projects such as the pan-Canadian Terry Fox PROFYLE research project (for patients aged 0–29 years who have poor prognosis cancers), the SickKids Cancer Sequencing Program (KiCS) in Ontario, the Personalized OncoGenomics (POG) program in British Columbia and the TRICEPS and SIGNATURE programs in Quebec. In addition, the ongoing CANTRK Ring Study aims to establish concordance at 17 sites across Canada for IHC and NGS testing for *NTRK* gene fusions [31]. Finally, *NTRK* gene fusion testing is available through Bayer’s complimentary *Fast*TRK clinical testing program (fasttrk.ca) and privately through companies such as Foundation Medicine (foundationmedicine.ca).

## 2. Method to Achieve Consensus on TRK Fusion Cancer Algorithms

In early 2018, a group of Canadian experts including medical oncologists, endocrinologists, pathologists, and molecular laboratory directors was assembled. Between 2018–2019, a series of consultancy meetings were held, during which the authors-developed draft algorithms for testing and treatment of TRK fusion-positive solid tumours. These tumour-specific algorithms were then used as the basis for discussion and further refinement. Consensus on these algorithms was reached through a series of teleconferences and emails. The algorithms and draft text were subsequently revised and recirculated through an iterative process until all authors agreed with and signed-off on the final content.

While this publication focuses on three pediatric tumour types (non-rhabdomyosarcoma [RMS] STS/unspecified spindle cell tumours, including IFS; differentiated thyroid carcinoma (DTC); and glioma), investigators have identified *NTRK* gene fusions in multiple additional pediatric tumour types in the larotrectinib trials [52,53]. There is a high unmet need for patients with locally advanced or metastatic TRK fusion cancer in all solid tumour types. In this publication, we aim to provide a Canadian consensus on how to identify and treat pediatric patients with TRK fusion cancer. Where possible, evidence to support estimated cases per year in Canada and proportions of patients who might undergo *NTRK* gene fusion testing, is included either from the literature or from clinical experience gained through Canadian efforts to establish an *NTRK* testing program. Currently, our group believes there are not sufficient data to support the use of one TRK-inhibitor over another giving the small cohorts of pediatric patients and relatively short follow-up for both drugs. More mature data are needed before making formal recommendations based on response rate and side effects.

## 3. Non-Rhabdomyosarcoma (RMS) Soft Tissue Sarcoma (STS)/Unspecified Spindle Cell Tumours

### 3.1. Background

Soft tissue sarcomas are a heterogeneous group of tumours arising from mesenchymal cells (hematopoietic and connective tissue). There are over 50 different sub-types of STS, with RMS occurring most frequently in childhood [54,55]. From 2013–2017 in the United States, the incidence of STS was 1.1 per 100,000 in patients aged 0–19 [56]. In Canada, STS as a whole had the second poorest 5-year survival of childhood cancers at 71%, with a 20.4% 5-year relapse rate [57]. To the best of our knowledge, no *NTRK* gene fusions have been identified in RMS, so we focus instead on non-RMS STS and unspecified spindle cell tumours. 

Histological/immunohistochemical features suggestive of a high probability of an *NTRK* gene fusion include: IFS-like, lipofibromatosis-like neural tumour, malignant peripheral nerve sheath-like tumour with stromal and perivascular hyalinization, S100 and CD34 co-immunoreactivity, SOX10 immunonegative, and adult-type fibrosarcoma [45]. Pan-TRK immunoreactivity is not completely specific, and nuclear/cytoplasmic staining is indicative of an *NTRK3* gene fusion [45,58]. However, cytoplasmic staining pattern suggests either physiologic TRK expression, *NTRK* upregulation or an *NTRK1*, *2* gene fusion [45,58,59]. 

Infantile fibrosarcoma is a type of STS occurring in children <2 years of age (and occasionally up to 5 years of age) [60]. It is similar to adult fibrosarcoma, but with a less aggressive course and a high rate of *ETV6-NTRK3* gene fusion (in 70–100% of cases) [47,60,61,62,63,64]. Generally, childhood fibrosarcomas, peripheral nerve sheath tumours, and other fibrous neoplasms have a favourable prognosis if localized and resectable, with a 91.7% 5-year survival [57]. In the largest retrospective analysis of IFS (*n* = 56), surgical resection was the initial treatment for 68% of patients, with 39% requiring first-line chemotherapy due to incomplete resection or progression and 9% receiving second-line chemotherapy [65]. In 9% of patients, disfiguring amputations were required [65]. The response rate to chemotherapy was 75%, with a 68–71% response rate to VA (vincristine, actinomycin-D) alone [65,66]. Combination chemotherapy regimens have been adapted from the adult setting and include: ifosfamide, cyclophosphamide, vincristine, actinomycin-D, etoposide, and others. In our experience, VA (with or without cyclophosphamide) is the most common first-line regimen in Canada [66]. Regimens containing alkylating agents may have significant acute toxicities and late effects, including risks of infertility and second malignant neoplasms [65].

Similar to IFS, for other non-RMS STS, surgical resection with adjuvant chemotherapy for patients with incomplete resection or high-risk features is the standard of care [67]. Combination chemotherapy regimens include similar agents as for IFS. In our experience, doxorubicin and ifosfamide, doxorubicin and cisplatin, and VAC are common first-line options in Canada, depending on histology. In the EpSSG NRSTS 2005, prospective, non-randomized study of 138 pediatric patients with STS, the response to chemotherapy was 55.2% [68].

In the Phase I/II larotrectinib trials, 28 pediatric patients with TRK fusion-positive IFS had an ORR of 96% with the median DOR NE [15]. In total, there were 48 pediatric patients with sarcoma (including IFS, STS, gastrointestinal stromal tumours and bone sarcoma) [69]. They demonstrated an ORR of 94% [69]. The median DOR in these 48 pediatric patients combined with 23 adult patients with STS was NE [69]. There were two patients with IFS in STARTRK-NG, both of whom responded to entrectinib [23].

### 3.2. Testing Consensus

The diagnostic workup for IFS includes FISH, reverse transcription polymerase chain reaction (RT-PCR), or NGS assay for the canonical *ETV6-NTRK3* gene fusion, at the discretion of the testing lab. Up to 30% of patients with IFS may be negative for the *ETV6-NTRK3* gene fusion and other *NTRK*, *BRAF*, and *MET* gene fusions have been identified in IFS [48,63,70,71]. Thus, we recommend an NGS panel including *NTRK1-3* gene fusions for patients who test negative for *ETV6-NTRK3* fusions. With five average annual pediatric cases of IFS, we estimate 0–2 patients/year in Canada will be negative for *ETV6-NTRK3* [47,60,61,62,63,64,72].

**Figure 1:**
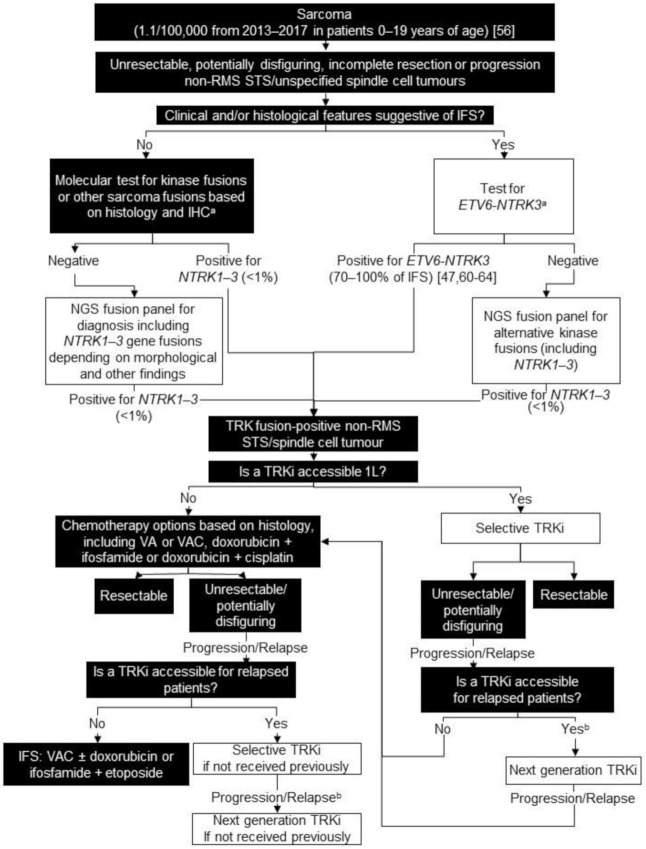
Biomarker testing and treatment for neurotrophic tyrosine receptor kinase (*NTRK*) gene fusions in sarcoma. White boxes with black outlines represent either *NTRK* gene fusion testing or treatment with a TRKi. Black boxes with white text indicate all other steps that do not include either NTRK gene fusion testing or treatment with a TRKi. ^a^ At discretion of lab; ^b^ Consider re-biopsy and molecular test to confirm acquired resistance mutation. ETV6 = ETS variant transcription factor 6; IFS = infantile fibrosarcoma; IHC = immunohistochemistry; L = line; NGS = next generation sequencing; RMS = rhabdomyosarcoma; STS = soft tissue sarcoma; TRK = tyrosine receptor kinase; TRKi = TRK inhibitor; VA = vincristine and adriamycin; VAC = vincristine, adriamycin, and cyclophosphamide. Knezevich et al. 1998 [47]; National Cancer Institute [56]; Knezevich et al. 1998 [60]; Bourgeois et al. 2000 [61]; Rubin et al. 1998 [62]; Sheng et al. 2001 [63]; Loh et al. 2002 [64].

For all non-RMS STS and unspecified spindle cell tumours, we recommend FISH, NanoString, or targeted NGS panels for known sarcoma fusions based on histology and immunohistochemical findings. At some institutions, this panel may include *NTRK1-3* gene fusions. Patients who are negative for known fusions should be offered an NGS panel including *NTRK1-3* gene fusions if not included in the original panel (Figure 1). As only an average of 25 children <15 years of age are diagnosed annually with fibrosarcoma or other non-RMS STS in Canada, proceeding directly to RNA-based molecular testing may be feasible [72]. We believe upfront testing for *NTRK* gene fusions in non-RMS STS is worthwhile because TRK inhibition may represent a reasonable first-line therapy.

### 3.3. Treatment Consensus

As *NTRK* gene fusions are pathognomonic in IFS, we recommend considering a selective TRK inhibitor as the first-line systemic treatment for unresectable or metastatic IFS. These agents have demonstrated impressive response rates in IFS with the potential to prevent disfiguring surgery, such as limb amputations, and to avoid cytotoxic chemotherapy in very young patients.

In locally advanced/unresectable or metastatic TRK-fusion positive non-RMS STS or in patients who would otherwise require a disfiguring surgery, we also recommend considering a selective TRK inhibitor as the first-line systemic treatment. The historically poor response to standard chemotherapy in non-RMS STS makes the impressive efficacy and tolerability of TRK inhibitors and the potential to prevent significant morbidity associated with surgery encouraging. However, further data are required to fully characterize the role of TRK inhibitors in non-RMS STS (Figure 1). The Children’s Oncology Group is currently running a Phase II trial exploring the efficacy of larotrectinib for previously untreated TRK fusion positive solid tumours (ADVL1823, NCT03834961).

## 4. Differentiated Thyroid Carcinoma (DTC)

### 4.1. Background

Thyroid carcinoma was the most common form of cancer in adolescents and young adults 15–29 years of age in Canada in 2017, comprising 16% of all cancer in this population [73]. From 2013–2017 in the United States, the incidence of thyroid carcinoma was 1.1 per 100,000 in patients aged 0–19 [56]. The majority (~93%) of thyroid carcinomas in children are DTC (papillary and follicular), ~5% of cases are medullary (MTC), and ~2% are a mix or rare forms [74]. 

Several recent studies have demonstrated that the molecular landscape of pediatric DTC differs from that in adults [75,76,77]. Although the prevalence of *NTRK* gene fusions in pediatric tumours varies among studies, it appears to exceed that in adults and may be as high as 26% [78]. 

Differentiated thyroid carcinoma is frequently more advanced at diagnosis in pre-pubertal children when compared to adolescents and adults, although the long-term prognosis is highly favourable for both children and adolescents [79,80,81]. 

Management guidelines generated by the American Thyroid Association guide the care of children and adolescents with DTC [82]. Surgery is the primary treatment modality with a sub-group of individuals meeting indications for radionuclide therapy. The American Thyroid Association has proposed three risk levels to predict likelihood of persistent/recurrent disease and to direct post-operative staging and adjuvant therapy: low risk, intermediate risk, and high risk [82]. 

Children with DTC generally have well-differentiated disease and respond well to first-line therapies (surgery +/− adjuvant radioactive iodine), thus systemic therapy is rarely indicated. We suggest consideration of systemic therapy for radioactive iodine refractory (RAIR) disease that is either symptomatic and/or progressive. While RAIR disease has not been specifically defined for a pediatric population, the definitions proposed by the European Thyroid Association and Cabanillas et al. are a reasonable starting point [83,84].

In addition, for the rare child with inoperable DTC, or for those wherein high surgical morbidity is anticipated, Kazahaya and colleagues have demonstrated a role for neo-adjuvant targeted therapy [85]. Robust data are lacking regarding systemic therapy options for pediatric patients with DTC. Among 28 patients with TRK fusion-positive thyroid carcinoma treated with larotrectinib in a pooled sub-analysis (*n* = 2 <18 years of age) the ORR was 75% with median DOR NE [86]. The American Thyroid Association guidelines recommend consultation with specialist centres and suggest a clinical trial as the preferred option, followed by an oral kinase inhibitor if a trial is not available [82].

### 4.2. Testing Consensus

We propose that for children presenting with advanced disease in the pre-operative setting (invasive cervical disease, bulky lymphadenopathy, or evidence of metastasis to the lungs, bones, or other distal sites), efforts should be advanced to preserve snap-frozen surgical specimens, as these will provide the most useful substrate for subsequent molecular analysis, should targeted therapy eventually prove necessary. This may require educating surgeons and pathologists about the need to bank tissue, as they are most often responsible for managing patients in the perioperative period.

**Figure 2:**
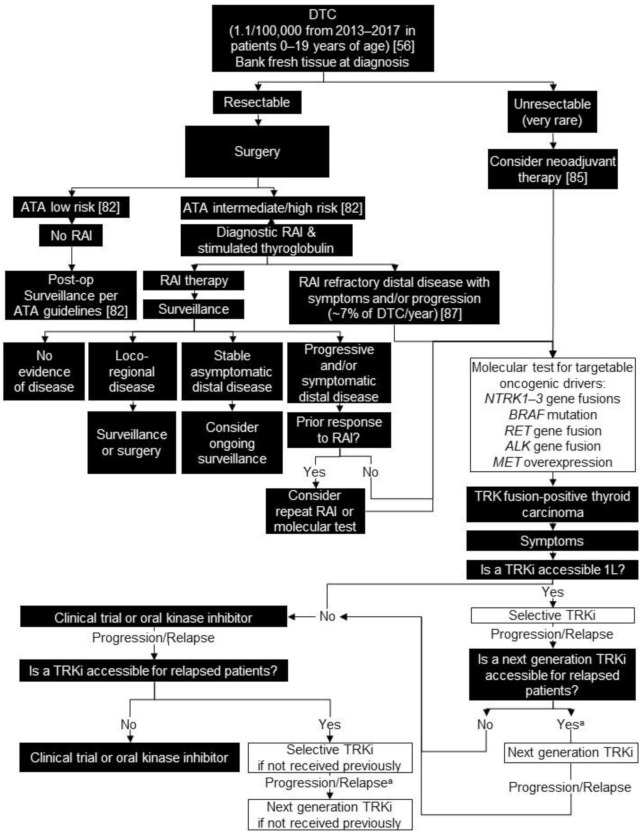
Biomarker testing and treatment for neurotrophic tyrosine receptor kinase (*NTRK*) gene fusions in differentiated thyroid cancer. White boxes with black outlines represent either *NTRK* gene fusion testing or treatment with a TRKi. Black boxes with white text indicate all other steps that do not include either NTRK gene fusion testing or treatment with a TRKi. ^a^ Consider re-biopsy and molecular test to confirm acquired resistance mutation. ALK = ALK receptor tyrosine kinase; ATA = American Thyroid Association; BRAF = B-Raf proto-oncogene, serine/threonine kinase; DTC = differentiated thyroid carcinoma; L = line; MET = MET proto-oncogene, receptor tyrosine kinase; RAI = radioactive iodine; RET = ret proto-oncogene; TRK = tyrosine receptor kinase; TRKi = TRK inhibitor. National Cancer Institute [56]; Francis et al. 2015 [82]; Kazahaya et al. 2020 [85]; Schmidt et al. 2017 [87].

We recommend comprehensive molecular testing by NGS (either whole-transcriptome sequencing or targeted RNA-based panels) in all patients with unresectable or progressive and/or symptomatic distal disease that is unresponsive to standard therapy of surgery and radioactive iodine (estimated 7% of cases/year) [87]. This testing should include targetable oncogenic drivers including: *NTRK1-3* gene fusions, *BRAF* mutations, *RET* gene fusions, *ALK* gene fusions, and *MET* overexpression/fusion (Figure 2).

### 4.3. Treatment Consensus

For those pediatric patients with TRK fusion-positive DTC who meet clinical criteria for systemic therapy, we recommend a selective TRK inhibitor, if accessible on a clinical trial, through public or private funding or compassionate access. We recommend this as first-line systemic therapy for patients who have failed standard therapy or who otherwise have contraindications to standard therapy, as TRK inhibitors have demonstrated good efficacy compared with standard of care options and are well tolerated (Figure 2).

## 5. Glioma

### 5.1. Background

Primary CNS tumours are the most common pediatric solid tumour and the most common cause of death from childhood cancer [88,89]. From 2013–2017 in the United States, the incidence of brain and other nervous system cancers in patients aged 0–19 was 3.2 per 100,000 [56]. Pediatric gliomas, including pilocytic astrocytoma, are the most common type of pediatric brain tumour [90,91]. 

In whole genome sequencing analyses of patients with LGGs, *NTRK* gene fusions have been found in 0.4–3.1% of tumours and were mutually exclusive with *BRAF* mutations and fusions as well as *NF1*, *MYB*, *FGFR1*, and *MYBL1* mutations [92,93]. Rearrangement driven LGGs are more likely in younger patients [92]. 

In general, pediatric LGGs have a good prognosis, if resectable [90,91]. However, they can be associated with significant morbidity depending on location [90,91,93]. In a Canadian Phase II study of vinblastine monotherapy in children with progressive LGG, the ORR was 25.9% and the disease stabilization rate was 87% [94]. In a study of children with progressive LGG, patients treated with an induction regimen of vincristine and carboplatin had an ORR of 57% and an 86% disease stabilization rate [95].

The association between tumour grade and outcome is less predictable in infants (<1 year of age); infant LGGs show a more aggressive course, while infant HGGs have a better outcome when compared with older children and adults [96]. However, some infant HGGs still have a particularly poor prognosis [97]. Anatomic location and infiltration often make surgical resection difficult, radiotherapy is not feasible due to its devastating neurocognitive impact, and standard systemic treatments have failed to show a significant impact on survival [97]. Pediatric HGGs are usually not the result of transformation from LGG and, in contrast to adult HGG, most commonly harbour recurrent mutations in the genes encoding histone H3.3 and H3.1 [98,99]. In a whole genome sequencing analysis of 127 pediatric patients with HGG, *NTRK1-3* gene fusions were identified in 10% of HGG and 4% of diffuse intrinsic pontine gliomas [100]. In studies of young children with HGG, 40% under 3 years of age and 16% under 4 years of age, had an *NTRK1-3* gene fusion [100,101]. *NTRK1-3* gene fusions are mostly found in hemispheric tumours, typically involve *NTRK2*, and have an intermediate prognosis [96,102]. 

Most of the systemic treatments studied for HGG provide limited improvement in survival [103]. Systemic therapy is often used to postpone or eliminate the need for radiation therapy, which can have debilitating long-term effects such as neurocognitive impairment, developmental delay, and endocrine abnormalities. Standard approaches for pediatric LGGs (vinblastine or vincristine/carboplatin) and HGGs (surgery, radiation, and alkylator-based chemotherapy) might not be adequate for TRK fusion-positive glioma. 

Twenty four (20 of whom were pediatric) with TRK fusion-positive primary CNS tumour were treated with larotrectinib in the SCOUT and NAVIGATE trials (13 high-grade glioma [HGG], 5 low-grade glioma [LGG], and 3 others) [20]. The ORR was 29%, median DOR was 4.9 months, median PFS was 11 months, and median OS was NE [20]. As of 1 July 2019, five patients with TRK fusion-positive primary CNS tumours were treated with entrectinib in STARTRK-NG and achieved an ORR of 80% [23]. The median DOR was not reported for patients with TRK fusion-positive primary CNS tumours [23]. Preclinical data suggest that entrectinib has good blood-barrier penetration but there are currently few in vivo data available for both drugs [104].

### 5.2. Testing Consensus

**Figure 3:**
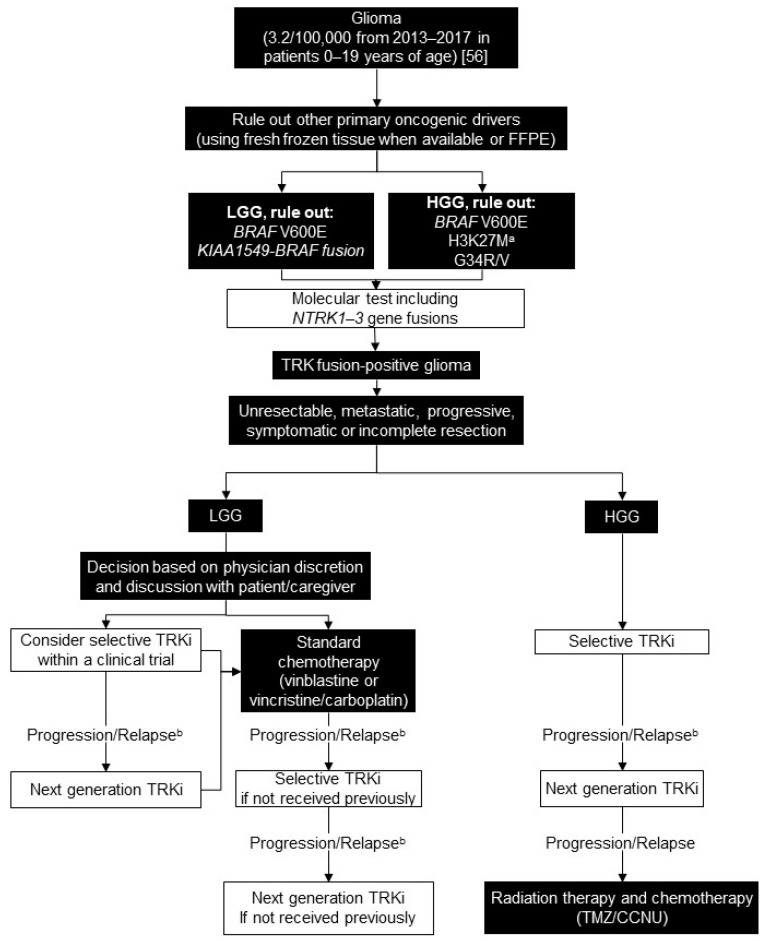
Biomarker testing and treatment for neurotrophic tyrosine receptor kinase (*NTRK*) gene fusions in glioma. White boxes with black outlines represent either *NTRK* gene fusion testing or treatment with a TRKi. Black boxes with white text indicate all other steps that do not include either NTRK gene fusion testing or treatment with a TRKi. ^a^ Rare cases of *NTRK* fusion and *H3K27M* mutation have been described; ^b^ Consider re-biopsy and molecular test to confirm acquired resistance mutation. BRAF = B-Raf proto-oncogene, serine/threonine kinase; FFPE = formalin-fixed paraffin-embedded; HGG = high grade glioma; IHC = immunohistochemistry; LGG = low grade glioma; TMZ/CCNU = temozolomide and lomustine; TRK = tyrosine receptor kinase; TRKi = TRK inhibitor. National Cancer Institute [56].

We recommend testing for common oncogenic drivers based on patient age, tumour location, and histology: *BRAF V600E* and *KIAA1549-BRAF* fusion for LGG and *BRAF V600E*, *H3K27M*, and *G34R/V* for HGG. Patients who are pan-negative for other primary oncogenic drivers should be tested for other molecular alterations including *NTRK1-3* gene fusions using RNA sequencing or NanoString. *NTRK1-3* fusions are particularly important to rule out in infants with hemispheric HGG. Pan-TRK IHC is not feasible due to native TRK expression in CNS tissue (Figure 3) [33].

### 5.3. Treatment Consensus

We consider systemic therapy in patients with unresectable, metastatic, progressive, or symptomatic LGG. A reasonable approach would be to treat with either standard chemotherapy (vinblastine or vincristine/carboplatin) or consider a selective TRK inhibitor as first systemic therapy within the context of a clinical trial. Should physicians choose to treat with standard chemotherapy first-line, we recommend a selective TRK inhibitor in patients who require a second-line systemic therapy (Figure 3).

Given the poor outcome and morbidities associated with non-targeted systemic therapy, we recommend considering a selective TRK inhibitor as the first-line systemic treatment in HGG with *NTRK* gene fusions that are unresectable, metastatic, progressive, or in some cases of localized HGG. This decision depends on the particular histology and whether there are any satisfactory alternatives. As more data becomes available, it will be interesting to see if one TRK inhibitor will show a better response rate, and tolerability. 

## 6. Tumour-Agnostic

### 6.1. Background

Among pediatric tumour types, IFS [36,61,105] and cellular and mixed congenital mesoblastic nephroma [36,46,47,48] harbour *ETV6-NTRK3* gene fusions at a frequency of >80%. Papillary thyroid cancer and pediatric HGG have an intermediate frequency of *NTRK* gene fusions (*NTRK1-3*) (5–25%), although the frequency of *NTRK* gene fusions in HGG increases to 40% in children under 3 years of age [36,78,100,101,106,107]. In LGG, *NTRK* gene fusions are found at a low frequency (<5%) [92,93].

Rosen and colleagues used a database of ~26,000 prospectively sequenced patients to identify those with *NTRK* gene fusions and assess their outcomes [13]. In 76 patients with TRK fusion cancer, the median PFS on first-line therapy (excluding TRK inhibitors) was 9.6 months and the ORR was 46.7% [13]. This illustrates that TRK fusion cancer can respond to standard of care, but not with a high ORR [13].

### 6.2. Testing Consensus

For cases with a high probability of harbouring an *NTRK* gene fusion, a FISH or RT-PCR test is standard if an *ETV6-NTRK3* fusion is suspected. Positive results would confirm the *ETV6-NTRK3* gene fusion and patients with negative results should be offered an RNA-based NGS panel for alternative oncogenic drivers, including *NTRK1-3* gene fusions, *BRAF*, and *MET*.

**Figure 4:**
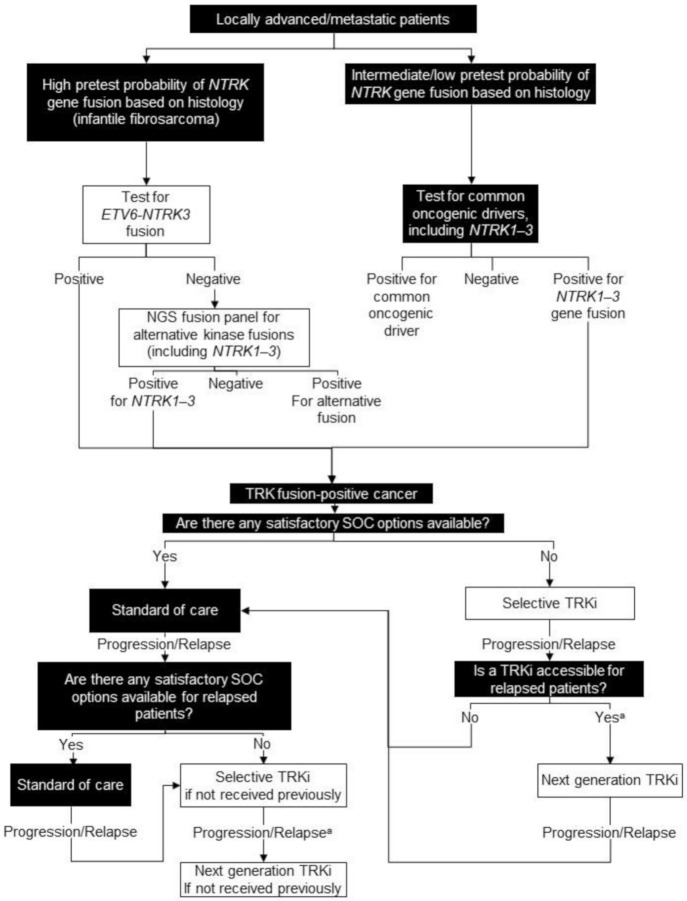
Biomarker testing and treatment for neurotrophic tyrosine receptor kinase (*NTRK*) gene fusions. White boxes with black outlines represent either *NTRK* gene fusion testing or treatment with a TRKi. Black boxes with white text indicate all other steps that do not include either NTRK gene fusion testing or treatment with a TRKi. ^a^ Consider re-biopsy and molecular test to confirm acquired resistance mutation. NGS = next generation sequencing; SOC = standard of care; TRK = tyrosine receptor kinase; TRKi = TRK inhibitor.

For tumour types with an intermediate or low probability of harbouring an *NTRK* gene fusion, ideally all patients with locally advanced/metastatic disease or those being considered for systemic therapy would be offered a comprehensive RNA-based NGS panel for all known oncogenic drivers, including *NTRK1-3* gene fusions (Figure 4).

### 6.3. Treatment Consensus

If there are standard of care treatment options considered satisfactory, we recommend exhausting these before treating a patient known to harbour an *NTRK* gene fusion with a TRK inhibitor, in accordance with the Health Canada-approved label. In areas of high unmet need, where none of the available options are considered satisfactory (e.g., surgery with high morbidity, significant toxicity, or low response rates), it is reasonable to consider a TRK inhibitor as the first systemic treatment in patients with TRK fusion cancer, ideally on study if available (e.g., NCT02637687 or NCT03213704) (Figure 4).

## 7. Regulatory Landscape of TRK Inhibitor Therapy in Canada

Larotrectinib was the first tumour-agnostic agent approved by Health Canada and the first molecularly-targeted therapy to be simultaneously developed and approved in adult and pediatric populations [28]. Entrectinib was approved shortly after. This represents a major paradigm shift in cancer treatment in Canada, ushering in an era of precision oncology for pediatric patients. Embedded in these approvals are a stipulation to provide post-market safety monitoring data to confirm the real-world applicability of the Phase I/II results. 

Phase III trials may not be possible to undertake for a common oncogenic driver found across heterogenous histologic subtypes. A new framework is required to assess efficacy of histology-agnostic, molecularly targeted clinical trials in order to ensure timely access to effective new therapies for all patients in need.

## 8. Conclusions

This consensus is intended to offer general principles on testing for *NTRK* gene fusions and treatment of pediatric patients with non-RMS STS/unspecified spindle cell tumours including IFS, DTC, and glioma. We also propose a tumour-agnostic consensus based on the probability of the tumour harbouring an *NTRK* gene fusion. This consensus is intended to offer general principles and should be adapted according to the histology and the testing methods/procedures available at each individual solid tumour biomarker lab, at the discretion of the pathologist and molecular lab director.

The TRK inhibitors have demonstrated favourable response rates and acceptable tolerability across tumour types in pediatric patients with TRK fusion cancer. For children with TRK fusion cancer, who have exhausted other treatment options or when there are no satisfactory treatment options, TRK inhibitor therapy should be considered as an effective option. However, since the optimal duration of treatment and long-term safety are unknown it will be essential to take into account this data as it becomes available, in addition to more mature data from ongoing clinical trials. 

We hope this consensus will assist healthcare professionals in identifying pediatric patients with TRK fusion cancer and treating these patients.
